# Examining the influence of technological self-efficacy, perceived trust, security, and electronic word of mouth on ICT usage in the education sector

**DOI:** 10.1038/s41598-024-66689-4

**Published:** 2024-07-13

**Authors:** Shuo Xu, Kanwal Iqbal Khan, Muhammad Farrukh Shahzad

**Affiliations:** 1 College of Economics and Management, Beijing University of Technology, Beijing, 100124 People’s Republic of China; 2 Department of Management Sciences, University of Engineering and Technology, New Campus, Kala Shah Kaku, Pakistan

**Keywords:** Intention to use for interaction, Technology self-efficacy, Electronic word of mouth, Actual use, Perceived trust, Perceived security, Intention to use for information, Environmental sciences, Environmental social sciences

## Abstract

The context of education has changed due to revolutionary developments in the information communication technology (ICT) industry in the post-COVID era. Innovative learning methods were introduced in the education sector to promote quality education. The students find it more convenient to use ICT tools to integrate their knowledge-seeking. China has recently paid more attention to developing and adopting electronic infrastructure. The study assesses the effect of technology self-efficacy (TSE) on ICT acceptance and implementation in China’s education sector. It also analyzed the role of perceived trust, perceived security, and electronic word of mouth (eWOM) in integrating digital information sharing and interaction tools. Data is collected from 382 business students at Chinese universities. The results revealed that perceived trust mediates the relationship between TSE and the actual use of ICT tools, intention to use ICT tools for information, and intention to use ICT tools for interaction. Further, perceived security and eWOM significantly moderate the relationship between TSE and perceived trust. The findings indicate that it is essential to offer assistance and instruction to students in the educational sector so they can use ICT technology more frequently. It is also crucial for organizations to establish a supportive culture and provide the necessary technological resources to facilitate the use of ICT.

## Introduction

Dependence on technology has rapidly increased over the years, especially in the post-COVID-19 era^1^. All industries have utilized advanced technology when lockdowns exist, and people interact minimally. Even after COVID-19, the fear remains, and people still rely more on advanced technological applications for learning^2^. The education sector is flourishing due to digitalization, the introduction of more compelling devices, and the utilization of new tools^3^. Many countries restructured their systems to adopt information communication technology (ICT) in educational sectors, but China’s transformation and unprecedented growth are evident. China is leading the way in this digital revolution in education and is a global leader in innovation and technology. Government initiatives, technological improvements, and the growing need for a more contemporary and effective educational system have pushed the acceptance and usage of ICT tools in Chinese educational institutions^4^. Integrating innovative technologies and constructing electronic infrastructure can open new avenues for students to learn. Knowledge transfer and sharing have become easy using ICT tools in education. The students can utilize different digital tools for learning purposes^5^.

The success of e-learning programs largely depends on the user’s acceptance of the system. It is based on students’ willingness to adopt digital tools for attaining knowledge and interaction purposes^6^. The individual acceptance of new technologies has remained a widely researched topic for the last two decades^7^. The technological acceptance model (TAM) is a fundamental behavioural model that explains the users' perspective on adopting and applying new technologies^8^. The theory of reasoned action (TRA) explains the user attitude that reshaped their behaviour and subjective norms that convert into the intention and later lead to actual actions using ICT tools^9^. TAM and TRA provide theoretical support to the digitalization process in Chinese education. Students have technological self-efficacy (TSE), increasing their intention to use modern technologies. Students perceived that the new techniques could polish their skills and provide ease and flexibility in learning, and they intend to adopt them^10^.

TSE is discussed earlier in technological applications like computers, mobiles, tablets, laptops, robotics, and the internet^11^. However, its implications in educational sectors to know more about its actual use or perceived use for interaction and information still requires more instigation^12^. Perceived trust is crucial in online platforms, increasing consumer behavioural intentions. It increases the customers’ satisfaction level, which leads to expanded use of ICT tools^4^. Students learn from online platforms when perceived trust is there. It also increases their intention to revisit and enhances the benefits of ICT in the education sector. The mediating role of perceived trust between TSE and the actual use of ICT tools, intention to use ICT tools for information, and intention to use ICT tools for interaction by Chinese university students needs more research.

Security concerns relate to the perceived risks associated with using ICT tools, such as data breaches, privacy issues, and cyber threats. The students often face online scams hacking and find inappropriate content and material, which reduces their trust levels in online platforms^13^. Scholars suggested that users' privacy should be protected through verification, passcode protection, encryption, and authentication^14^. The security increases consumers' trust and behavioural perceptions. When consumers know their personal information is not used elsewhere, their trust level will be high on the website^15^. A higher level of TSE often correlates with increased confidence in using ICT tools, leading to more innovative and effective educational practices. It is important to determine how students engage with ICT tools, innovate teaching methods, and adapt to technological advancements.

Electronic word of mouth (eWOM) refers to sharing opinions, experiences, and recommendations about ICT tools and platforms through online platforms and social networks. Positive eWOM can enhance ICT tools’ perceived usefulness and credibility, encouraging their adoption and use among educators and students^16^. TSE increases students' perceived trust level, but positive eWOM further strengthens the relationship. The moderating role of perceived security and eWOM between TSE and perceived trust has rarely been studied. That is why the study aims to analyze the impact of TSE on actual use and intention to use ICT tools for information and interaction by Chinese students through perceived trust. It also explains the moderating role of perceived security and eWOM between TSE and perceived trust. The findings will contribute to the Chinese educational sector and present the students' behavioural intention to accept and implement ICT for learning and knowledge-sharing purposes. It also helps policymakers devise policies to promote e-learning technologies and make them more secure by keeping user concerns in mind.

## Theoretical underpinning and hypotheses development

The theory of planned behaviour extends the TRA, which discusses individuals’ actual and perceived behaviour toward technology adoption. These theories are based on the Belief-Attitude-Intention (B-A-I) model^17^. It implies individual perceived behaviour, cultural norms, and situational factors as predictors of their actions. It also explains the individuals' behaviour and subjective norms that can impact their intention, which later turns into the actual behaviour^7^. In the context of technology adoption in education, TRA refers to an individual’s positive or negative feelings about using ICT tools. If students believe they can effectively use technology (high TSE), they are more likely to have positive attitudes toward adopting ICT tools. These are perceptions about whether others think they should or should not use ICT tools^18^. eWOM can influence these norms. Positive reviews or recommendations from colleagues or peers can encourage technology adoption.

The application of ICT for learning is also an essential element of technology development models. The TAM strengthens the actual and perceived individual use of technological tools. Its two basic divers, utility and perceived ease of use, are the primary determinants of depicting the intentions to apply technology^18^. It was extensively involved in diversified fields due to its intelligibility in describing the attitudes of individuals toward advanced innovation and technology adoption^19^. TAM suggests that perceived trust is a key determinant of users’ attitudes towards using a technology. Trust in ICT tools is crucial for their adoption. Users need to believe that the technology will perform reliably and securely^8^. Security concerns can act as barriers to technology adoption. Users need assurance that their data and privacy will be protected when using ICT tools. Positive eWOM can influence attitudes and subjective norms, encouraging more educators and students to adopt ICT tools^20^. The study is based on the TRA and the TAM to answer how TSE affects the actual use of ICT tools, information, and interaction in the education sector.

### Technological self-efficacy and use of ICT tools

TSE depicts the individuals' behaviour regarding the adaptability of digital learning in the educational sector through traditional and advanced applications^21^. The students’ acceptance of online applications motivates them to learn through advanced technologies^7^. These could save their time, create ease, and provide flexible learning opportunities. Most have the knowledge and access to digital tools like tablets, laptops, computers, and mobile phones with internet access^22^. Therefore, it is not difficult for them to utilize the same for educational purposes. Studies show that students’ intention to use ICT tools in learning could enhance their satisfaction due to perceived ease and usage^23^. That is why the past study^24^ highlighted a positive relationship between TSE and the actual use of ICT tools. TAM represents the users’ intention towards technology acceptance and adoption. It provides theoretical grounding for the students' actual use of ICT and discusses their future intent to apply it for information and interaction.

### Mediating role of perceived trust

Trust is a specific attitudinal response of a person to another in exchange for fairness, truthfulness, and authenticity. All social relationships are based on trust, particularly in online platforms where advanced technology is applied; it is worth considering^25^. Both parties confidently deal with whether an element of trust exists. Many studies analyzed the role of consumer credibility and trust in online networks and demonstrated its persuasiveness across different scenarios^26,27^. It creates satisfaction, increasing individual intention to use the product or service again. The expectancy-disconfirmation theory states that one can be satisfied if the perceived outcomes exceed the expectation level, which can be more productive if the trust element exists^28^.

One key determinant influencing students’ adoption and usage of ICT is technology self-efficacy, which refers to an individual's trust in their ability to use technology to achieve desired outcomes. Higher levels of trust have been associated with increased motivation to learn and use technology, leading to more frequent and proficient ICT usage^19^. Perceived trust refers to an individual's confidence in technology’s reliability, security, and effectiveness. Cultural and societal factors may influence Chinese university students’ perceptions of trust in technology. China’s rapid technological advancements and emphasis on education may shape students' attitudes and beliefs toward technology, affecting their perceived trust and subsequent ICT usage patterns^4^. Students would be more likely to integrate technology into their teaching and learning procedures and feel more at ease. Customers are more likely to see technology favorably and perceive it as reliable, which might increase the acceptance and usage of ICT in education. Because regulatory agencies are seen as reliable, educational stakeholders might be more open to experimenting with new ICT tools and platforms^25^.

Customers will be more engaged and satisfied if they trust service providers^29^. This reflects that system authenticity and quality information are the main determinants of trust that increase the usage of online applications. Trust creates a lasting relationship in an online environment where one-to-one interaction is missing^30^. In an online venue, TSE can enhance ICT tools among Chinese students, but trust can increase their actual and perceived application for learning and knowledge sharing. Suppose a student intends to apply to an online education system. In that case, their trust will be high in such a learning mode that ultimately increases ICT's actual and intentional use for information and interaction. These arguments are hypothesized below:

*H*_*1*_ *: Perceived trust mediates the relationship between TSE and the actual use of ICT tools among Chinese university students.*

*H*_*2*_ *: Perceived trust mediates TSE and the intention to use ICT tools for information in the Chinese university.*

*H*_*3*_ *: The relationship between TSE and the intention to use ICT tools for interaction is mediated by perceived trust among Chinese business students.*

### Moderating role of perceived security

TSE describes the individual effort of learning digital tools to keep updated with the online environment. When students are convinced that new technologies can benefit them in their learning and knowledge-seeking process, they are ready to adopt them and learn new techniques to help them utilize these tools. It reflects their positive attitude towards e-learning. However, trust is a major obstacle to online systems^31^. Students often doubt whether they will visit a website due to the increasing number of online scams. Therefore, researchers suggested a secure system that enhances user trust^15^. When consumers are sure their personal information is not shared, stolen, or misused on any online platform, their trust level increases^32^. Worries about data breaches, unauthorized access, or abuse of personal information can greatly impact perceived security in an educational setting. The confidence people have in ICT is the belief that their data will be managed responsibly and securely and that the system will perform as expected. In this regard, a person with strong technology self-efficacy may become less trusting of ICT use if they have security worries. On the other hand, even among those who have a lesser level of technology self-efficacy, robust perceived security measures can increase trust. However, TSE increases the perceived trust. But, the intervening role of perceived security will highly strengthen the relationship. The same is posited below in the form of hypothesis 4.

***H***_***4***_ ***:*** *Perceived security moderations the relationship between TSE and perceived trust, such that the effect of TSE on perceived trust is more assertive when perceived security is higher.*

### Moderating role of electronic word of mouth

EWOM is an exchange of views, feedback and opinions among e-users, which can affect their revisit and reuse intention and behaviour. It is presented through word-to-word communication, comments on the website and social networking online^33^. EWOM is spread globally through emails, blogs, review sites, etc., due to the free Internet freedom of expression^34^. It plays a significant role in building the reputation of the online platform. Previous studies show that positive eWOM is a marketing tool that increases the sale, use, and value of products and services internationally and geographically^35,36^. A positive eWOM helps to establish a trustworthy positive relationship between students and educational institutes that increases the use of online platforms for learning^37^. TSE motivates the students to utilize their existing knowledge and develop new skills to apply ICT, increasing their perceived trust in e-learning. The sharing and consumption of knowledge have changed dramatically in the digital age. eWOM, which is frequently disseminated through review sites, social media, and online forums, has grown to be a significant factor in decision-making. Positive feedback and suggestions from peers, colleagues, or reliable sources can allay doubts and anxieties regarding the use of ICT in education, which in turn increases perceptions of the reliability of solutions offered by ICT. However, the eWOM makes this relationship stronger and more constructive due to the positive communication by the users. The same argument is presented in the below hypothesis:

*H*_*5*_ *: eWOM moderates the relationship between TSE and perceived trust, such that the effect of TSE on perceived trust is more robust when eWOM is positively increased.*

**Figure 1:**
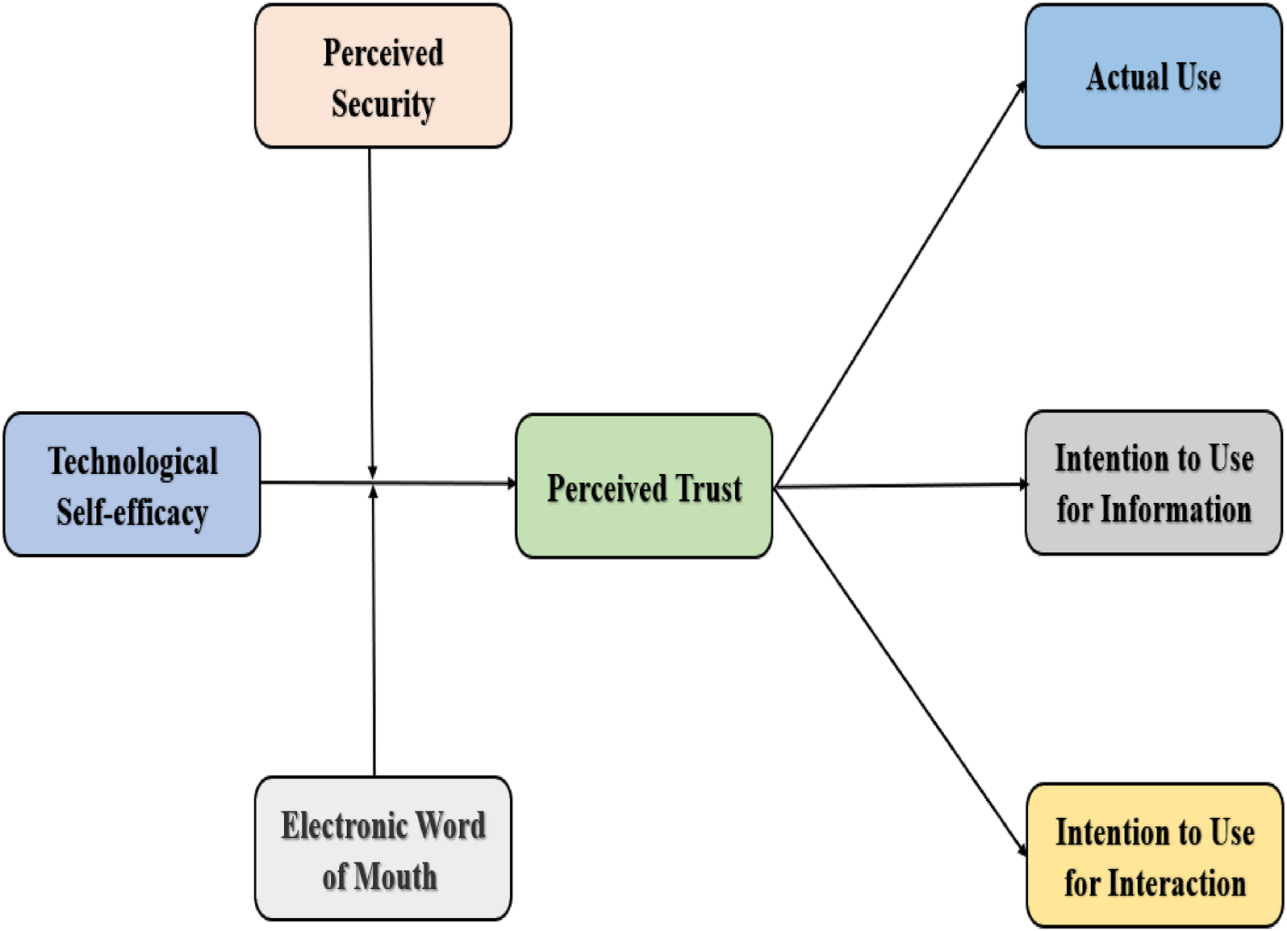
Theoretical framework.

Figure 1 explains the theoretical framework of the study.

## Research methodology

Many educational institutions have encouraged using ICTs for the sustainability of education. This study empirically investigated students’ intention to use ICT for information and interaction in their education’s long-term viability. Students who participated in blended learning and used ICT in their education made up the study sample. The survey’s questions regarding demographics were evaluated on a five-point Likert scale, with “1” representing strongly disagree and “5” representing strongly agree. The distribution of questionnaires employed self-administration, and respondents were requested to respond to questions about ICT use and its impact on their education. The collected data were scrutinized with the assistance of the PLS-SEM technique using Smart PLS 3.3 statistical software. Factor loadings were employed to establish construct validity and reliability for the model's satisfactory level of fit, as endorsed by^38^.

### Data collection and sample characteristics

The respondents were students from China’s standard universities, emphasizing developing and integrating ICT implementation and acceptance in China's education sector. Tablet computers are a common learning tool for ICT among university students from the campuses. For data gathering, we randomly selected six top-ranking universities in Beijing, China, as listed by Times Higher Education. The higher education ministry ranked the selected sample universities at the top based on the curriculum, learning objectives, and instructors. The eligible participants in this survey were referred to as the target population^39^. This extensive investigation gathered a demonstrative sample of the target population to deliver the essential data. Nonprobability sampling was used as a sampling frame that could not be created or acquired because it was difficult to get a list of potential respondents; convenience sampling was used. The sample size was calculated using the suggested method^40,41^. A comparable strategy was used by prior investigations^42,43^. The investigators shared information about the study constructs with participants via emails, WeChat, Weibo, and social media posts.

We are requesting that students voluntarily complete the questionnaire. We employed self-administered primary data collecting, both online and offline. Self-completed questionnaires were used in this survey method study to gather the primary data. This study used self-administered questionnaires since they speed up data collection from a larger sample size. Questionnaires were manually and electronically sent to qualified respondents to boost the response rate. The research team was approached on respective campuses to obtain authorization to gather study data and explain its goals. We have distributed a total of 520 questionnaires to university students. 406 participants were responded, with a response rate (78%). However, the data was further scrutinized as many questionnaires were incomplete. Some were invalid for data analysis due to mistakes, biasedness, and missing replies. After careful examination of the data, multivariate outliers, and unengaged responses of all returned questionnaires, leaving 382 useable cases for analysis. The final response rate is 73% of usable data. This sample size aligns with recommendations suggesting that empirical research should involve more than 30 but fewer than 500 participants^44^.

However, the sample data contains 211 male (55%) and 171 female (45%) respondents. The age of respondents is categorized as the maximum, with 179 respondents (47%) between 27 and 33 years old. 102 (27%) were lied in 20–26 years old, while the remaining 101 (26%) were above 33. Moreover, the educational background of respondents consists of the following distribution: 194 respondents (51%) had bachelor's degrees, 107 (28%) had master's degrees, and 81 (21%) were Ph.D. doctors. The participants' educational background consists of 141 (37%) belonging to management science and 118 respondents (31%) belonging to business administration. 81 participants (21%) belonged to hotel management. 42 respondents (11%) belonged to other fields. Furthermore, the experience of respondents with ICT consists of the following distribution: 53 respondents (14%) have low experience with ICT, 188 (49%) have good experience with ICT in education, and 141 (37%) have high expertise to use ICT. Table 1 displays further respondent information.

**Table 1:** Respondent information.

Demographics	Distribution	n = 382
Gender	Male	211 (55%)
	Female	171 (45%)
Age group	20–26 years	102 (27%)
	27–33 years	179 (47%)
	Above than 33 years	101 (26%)
Education	Bachelor	194 (51%)
	Masters	107 (28%)
	PhD	81 (21%)
Specialization	Management Sciences	141 (37%)
	Business Administration	118 (31%)
	Hotel Management	81 (21%)
	Others	42 (11%)
Year of Study	First	78 (20%)
	Second	132 (35%)
	Third	109 (29%)
	Fourth	63 (16%)
Experience with ICT	Low	53 (14%)
	Good	188 (49%)
	High	141 (37%)

### Instrumentations

This questionnaire is divided into two parts. The first component of the survey consists of inquiries about the respondents' background, such as gender, age, and employment history. After filtering questions, respondents were asked whether they were enrolled in graduate/post-graduate programs at universities in China (See Table 1). Second section of the questionnaire was designed to measure the components employed in this investigation. All questions were modified from earlier research. The interval scale (Likert type) was used to collect participants’ responses. TSE in ICT is about individuals’ belief in their aptitude to leverage digital technologies for educational purposes, encompassing both the ability to utilize existing tools and the adaptability to learn and apply new ones effectively^10^. It was measured by seven items taken from^45^.

The actual use of ICT in education involves integrating technology into all aspects of the educational process, from content delivery and assessment to communication and administration^46^. It aims to create more engaging, efficient, and effective student learning and was assessed by five items taken from^47^. The intention to use ICT for information refers to an individual deliberate and conscious plan to incorporate ICT tools, resources, and solutions into the educational process to enhance information acquisition, dissemination, and utilization^6^. It was estimated that six items were taken from^48^. Intention to use ICT for interaction can be defined as the purposeful and planned commitment of educators, institutions, or individuals to incorporate ICT tools in the learning process to facilitate communication, collaboration, and student engagement^49^. It was measured by six items adopted by^50^.

Perceived trust refers to the subjective belief or confidence that individuals, such as students, have in the reliability, effectiveness, and ethical use of ICT technologies and digital information within the realm of education^51^. Six items of perceived trust were suggested by^25^. Perceived security refers to individuals' assessment or belief in ICT technologies used in educational settings. It encapsulates the collective perception of how well ICT infrastructure and practices safeguard sensitive data, ensure privacy, and mitigate risks associated with cyber threats and breaches within educational institutions^52^. It was evaluated by five items taken from^53^. eWOM is the digital dissemination and sharing of opinions, recommendations, reviews, and information about ICT tools, services, platforms, and practices used in educational settings^54^. It was assessed by three items taken from^55^. Details of measurements of items are attached in Table 2.

**Table 2:** Factors analysis.

Construct	Item		FL	α	CR	AVE	Sources
Technological Self-efficacy (TSE)				0.925	0.940	0.694	^45^
	TSE1	I have access to the internet, which I can utilize for my educational goals	0.887				
	TSE2	I can learn using a learning management system, such as ICT tools	0.896				
	TSE3	I am motivated to learn ICT-based technology	0.767				
	TSE4	ICT technology used for educational purposes has become customary	0.798				
	TSE5	I have to use ICT technology to study and revise my lesson	0.916				
	TSE6	It has become commonplace to use an ICT application	0.790				
	TSE7	I should find ICT technology applications more valuable than the requested sacrifice	0.761				
Perceived Trust (PTR)				0.897	0.921	0.662	^25^
	PTR1	I trust the security of using ICT	0.810				
	PTR2	I am sure technology and legal frameworks effectively shield me from online risks	0.843				
	PTR3	I believe that the government encourages the use of ICT	0.846				
	PTR4	I believe our government to be trustworthy; the government will respect my privacy	0.792				
	PTR5	I trust that ICT now has a safe environment, and we can perform ICT-related activities with our government	0.877				
	PTR6	I trust ICT is helpful in the preparation of my lessons and research	0.703				
Perceived Security (PS)				0.909	0.933	0.735	^53^
	PS1	I would be concerned about giving information to the ICT	0.899				
	PS2	I would be concerned that the information I give to the ICT system could be misused	0.827				
	PS3	I am concerned that my personal information, which I have provided for ICT purposes, could be misused	0.836				
	PS4	I would be concerned about giving information to the ICT	0.926				
	PS5	It would bother me if the ICT system asked me for personal information	0.794				
Electronic Word of mouth (eWOM)				0.883	0.905	0.613	^55^
	eWOM1	Using social media, the eWOM could increase the willingness to adopt ICT	0.756				
	eWOM2	I used social media, and eWOM could encourage people to use ICT	0.779				
	eWOM3	In education, the eWOM on multimedia plays a main role in increasing the intention to implement and adopt ICT in classrooms	0.840				
	eWOM4	I believe eWOM helps to use ICT technology in educational activities	0.792				
	eWOM5	I used to access eWOM about ICT tools and was satisfied	0.740				
	eWOM6	Online reviews and recommendations about ICT have been helpful to me	0.786				
Actual Use (AU)				0.856	0.896	0.634	^47^
	AU1	I believe that ICT technology is necessary for me to learn at any moment	0.748				
	AU2	To learn any place, I believe I must use ICT technology	0.818				
	AU3	Ad-hoc, irregular issues with learning are something I constantly struggle with	0.804				
	AU4	I believe that employing ICT Technology will enable me to continue learning wherever I am without interruption	0.807				
	AU5	I believe using ICT technology will offer learning activities and real-time services	0.802				
Intention to Use for Information (ITUINF)				0.873	0.902	0.604	^48^
	ITUINF1	I will consider using ICT	0.713				
	ITUINF2	I am planning to use ICT	0.793				
	ITUINF3	I will continue to use ICT	0.777				
	ITUINF4	I will inform others regarding the goodness of ICT	0.794				
	ITUINF5	I will use the resources essential to use ICT	0.798				
	ITUINF6	I pay reasonable attention to using ICT technology for my education	0.786				
Intention to Use for Interaction (ITUINT)				0.918	0.936	0.711	^50^
	ITUINT1	ICT technology used for educational purposes has become customary	0.875				
	ITUINT2	I have to use ICT technology to study and revise my lesson	0.876				
	ITUINT3	It has become commonplace to use an ICT application	0.853				
	ITUINT4	Utilizing ICT technology is simple	0.783				
	ITUINT5	Learning about computer-based technology is simple	0.860				
	ITUINT6	Interaction with ICT is simple and understandable	0.806				

Note: FL = Factor loadings, VIF = Variance inflation factor, CR = Composite reliability, AVE = Average variance extracted, α = Cronbach's alpha.

## Analysis and results

The PLS-SEM technique was used to analyze the data. This multivariate approach is frequently used to examine the correlation between many construct variables. The PLS-SEM technique was chosen because it is adaptable to small sample sizes and disregards normal data distribution. The statistical software SMARTPLS 3.3 version (https://www.smartpls.com/) was used for data analysis and model testing^56^. Measurement and structural assessment models were used to test the preliminary hypothesis as recommended by^57^. First, the measurement models have proposed construct reliability, convergent, and discriminant validity. The second method was a structural model, which provides the overall significance of the hypothesis. It improves statistical analysis’s effectiveness, simplicity, and precision. Compared to other analysis approaches, PLS-SEM is best suited for creating new, theoretically complex models that meet goals^58^.

### Measurement model

The SEM approach's measurement model had to be evaluated first to see if the data could be used for preliminary hypothesis testing^59,60^. The two processes in the measurement model procedure to choose the validation criteria are construct reliability and validity^61^. In the construct reliability procedure, Cronbach alpha, composite reliability, and AVE become the assessment benchmarks. The author stated that data with Cronbach alpha and CR values greater than 0.7 have strong internal consistency reliability. Similarly, AVE values higher than 0.5 show valid reliability of constructs. Additionally, for the observable variables to sufficiently clarify the latent variables, factor loadings must be higher than 0.7^62^. Factor loadings were employed to establish construct validity and reliability for the model’s satisfactory level of fit, as endorsed by^38^. The Cronbach alpha value for actual use = 0.856, intention to use for interaction = 0.918, intention to use for information = 0.873, TSE = 0.925, perceived trust = 0.897, perceived security = 0.909, and eWOM = 0.883. The CR value for actual use = 0.896, intention to use for interaction = 0.936, intention to use for information = 0.902, TSE = 0.940, perceived trust = 0.897, perceived security = 0.933, and eWOM = 0.905. The AVE value for actual use = 0.634, intention to use for interaction = 0.711, intention to use for information = 0.604, TSE = 0.694, perceived trust = 0.662, perceived security = 0.735, and eWOM = 0.613. As a result, the measurement model has strong convergent validity for data. The factor loading values for measurement models for university students are shown in Table 2.

**Figure 2:**
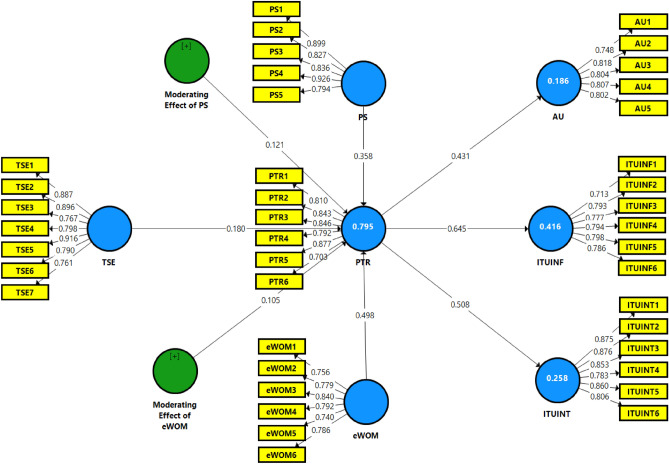
Measurement model (PLS Algorithm Diagram).

The Fornell-Larcker approach was used to evaluate the discriminant validity. The diagonal values in bold in Table 3 are the square root of the AVE, which must be greater than the correlation value between the variables. The AVE value for data from university students in this study is greater than the correlation value between latent variables. However, HTMT is considered a superior method to Fornell-Larcker criteria because it discriminates more explicitly among constructs^63^. In the study, all the HTMT values are also within range and considered acceptable for discriminant validity. As a result, the proposed model can be explained by the discriminant validity, which is sufficient. See Fig. 2, which explains the measurement model's estimation.

**Table 3:** Discriminant validity.

Constructs	1	2	3	6	7	8	9
1. Actual Use	**0.796**						
2. Intention to Use for Information	0.464	**0.777**					
3. Intention to Use for Interaction	0.760	0.640	**0.843**				
6. Perceived Security	0.364	0.665	0.383	**0.858**			
7. Perceived Trust	0.431	0.645	0.508	0.757	**0.814**		
8. Technological Self-efficacy	0.419	0.612	0.452	0.836	0.764	**0.833**	
9. Electronic Word of Mouth	0.491	0.581	0.599	0.558	0.793	0.614	**0.783**

Note: Bold values are the square root of the AVEs.

### Structural model

**Figure 3:**
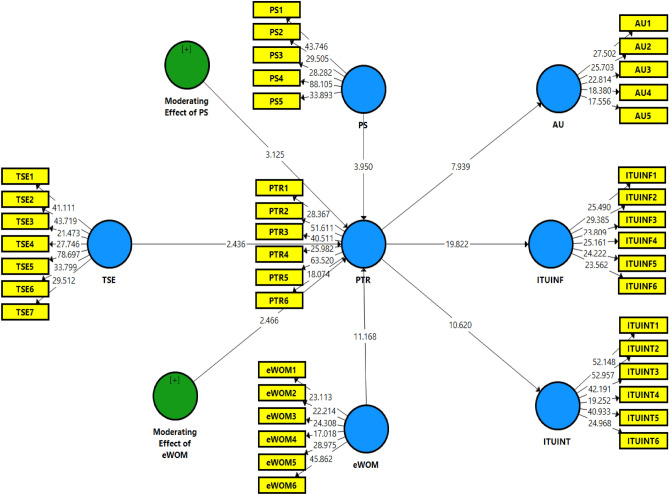
Structural model (PLS-SEM bootstrapping analysis).

Bootstrapping was used for the structural path’s significance assessment in the second step of evaluating the structure equation modeling. In the bootstrapping approach, sub-samples (5000) were assessed with replacements to look for errors, and the results guided the predicted t-values for the proposed model’s significance test^64^. According to^65^, the t-value must be considered to ascertain the association’s importance. This study demonstrates a significant connection using a t-value more than 1.96 and a p-value lower than 0.05. Furthermore, mean and standard deviation (SD) values can also be useful for assessing the normality of the data distribution. While PLS-SEM is often considered robust to violations of normality assumptions, understanding the data distribution can still be valuable for interpreting results and ensuring the reliability of the findings^66^. Figure 3 illustrates how the bootstrapping method approaches data normality for structural models. Thus, Table 4 presents the suggested model quality requirements. The variance inflation factor (VIF) in all constructs is used in the first phase to evaluate the multicollinearity in the study model. The VIF value should not exceed five to guarantee that the constructs have no multicollinearity issue. The model utilized in this study is sound based on the measurements, as indicated in Fig. 2, where the R^2^ value is high. The results demonstrate the value of R^2^ in the independent constructs, such as perceived trust, explains 18%, 41%, and 25% of the variation in AU, ITUINF, and ITUINT. The goodness of fit (GoF) index was also determined using the standardized root mean square residual (SRMSR) values for the constructs^67^. The values of SRMR = 0.135, which indicate that the model is a good fit. See Fig. 3, which explains the structural model's estimation.

**Table 4:** Hypothesis testing.

Relationships	β-values	Mean	SD	t-values	p-values	Results
Direct effects						
PS → PTR	0.358	0.346	0.091	3.928	0.000	Supported
PTR → AU	0.431	0.439	0.050	8.697	0.000	Supported
PTR → ITUINF	0.645	0.647	0.034	18.757	0.000	Supported
PTR → ITUINT	0.508	0.512	0.046	11.128	0.000	Supported
TSE → PTR	0.180	0.190	0.078	2.327	0.020	Supported
eWOM → PTR	0.498	0.501	0.042	11.852	0.000	Supported
Mediation effect						
TSE → PTR → AU	0.078	0.083	0.035	2.217	0.027	Supported
TSE → PTR → ITUINF	0.116	0.122	0.049	2.377	0.018	Supported
TSE → PTR → ITUINT	0.092	0.098	0.043	2.146	0.032	Supported
Moderation effect						
TSE*PS → PTR	0.121	0.121	0.042	2.872	0.004	Supported
TSE*eWOM → PTR	0.105	0.107	0.046	2.303	0.022	Supported

Note: ITUINT = Intention to Use for Interaction, TSE = Technological Self-efficacy, eWOM = Electronic Word of Mouth, AU = Actual Use, PTR = Perceived Trust, PS = Perceived Security, ITUINF = Intention to Use for Information, SD = standard deviation.

Table 4 presents the ultimate judgment on hypothesis development. This study's revealed the findings as it looked at the connection between TSE and perceived trust. The significant association was indicated by results (β = 0.180, t = 2.327, p < 0.05). This study examined the connection between perceived trust and actual use. The results showed a significant association (β = 0.431, t = 8.697, p < 0.05). This study examined how perceived trust and intention to use it for information are related. The significant association was indicated by results (β = 0.645, t = 18.757, p < 0.05). This study also examined how perceived trust and intention to use for interaction are related. The results indicated a significant association (β = 0.508, t = 11.128, p < 0.05).

For mediation analysis, the results of this study revealed that perceived trust positively mediates the relationship between TSE and actual use as values (β = 0.078, t = 2.217, p < 0.05). Perceived trust positively mediates the relationship between TSE and intention to use for information, as results indicated (β = 0.116, t = 2.377, p < 0.05). Furthermore, perceived trust positively mediates the relationship between TSE and intention to use it for interaction, as results indicate (β = 0.092, t = 2.146, p < 0.05). Thus, our study hypotheses H1, H2, and H3 are accepted. For moderation analysis, the results showed that perceived security positively moderates the relationship between TSE and perceived trust. The results indicated a significant association (β = 0.121, t = 2.872, p < 0.05). Thus, our study hypothesis H4 is accepted. Furthermore, eWOM positively moderates the relationship between TSE and perceived trust. The results indicated a significant association (β = 0.105, t = 2.303, p < 0.05). Thus, our study hypothesis H5 is accepted.

### Moderating graphs

**Figure 4:**
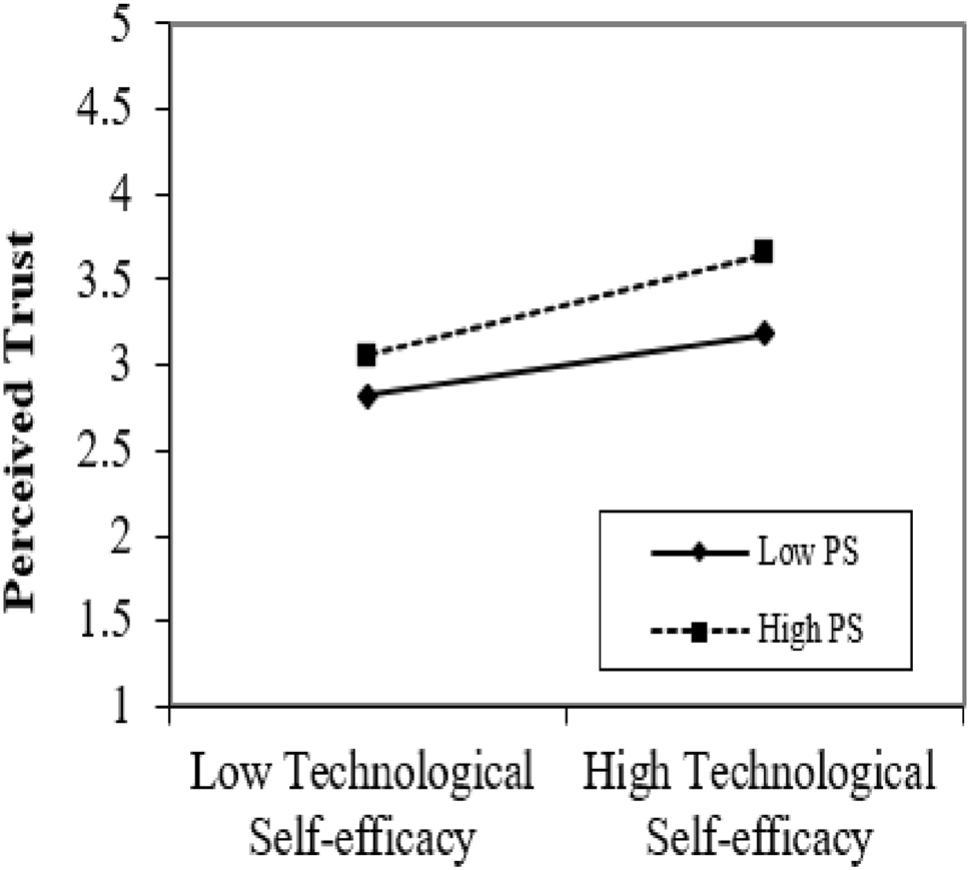
Moderating graph of PS.

**Figure 5:**
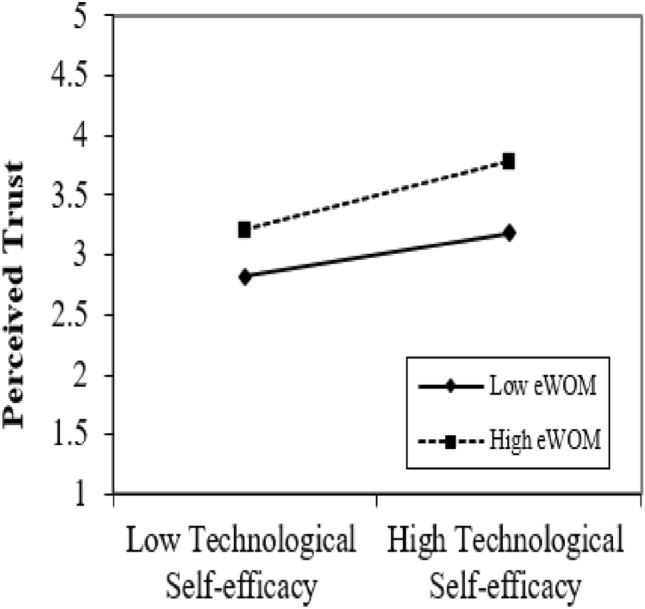
Moderating graph of eWOM.

We contrived the graphs to describe the results of moderating the role of perceived security and eWOM. Figures 4, 5 display the moderating role of perceived security and eWOM between perceived trust and TSE, respectively. Figure 4 depicts the moderating effect of perceived security between TSE and perceived trust. The vital point in the moderation graphs is that TSE and perceived trust are stronger under high perceived security. Figure 5 clarifies the moderating effect of eWOM on TSE and perceived trust. The prominent point in the moderation graphs is that TSE and perceived trust are powerful under high eWOM. This ultimately supports H4 and H5.

## Discussion

In the modern era, ICT has permeated every facet of human life, revolutionizing how we communicate, work, and learn. ICT integration has emerged as a powerful catalyst for transformation in education, offering novel avenues to enhance teaching and learning experiences^68^. China's education system is characterized by its sheer scale and complexity, with millions of students and educators spread across diverse geographical and sociocultural landscapes. The integration of ICT in this context is not a mere choice but a strategic imperative for fostering innovation, promoting equitable access to quality education, and preparing a globally competitive workforce^69^. Therefore, the current research study examines the impact of TSE on the intention to use ICT for information and interaction, as well as their actual use through perceived trust in the education sector of China. It also investigates the moderating role of perceived security and eWOM between TSE and perceived trust. A web-based questionnaire of 382 students from China's universities evaluates the conceptual framework. Five significant research findings are presented in the study.

Firstly, the findings state that perceived trust mediates the relationship between technological self-efficacy and the actual use of ICT tools. Students' trust in technology plays a crucial role as individuals believe in ICT technology's reliability, security, and effectiveness in their actual use. A past study^70^ suggested that students in the Chinese education sector have varying levels of confidence in their ability to use ICT technology for educational purposes. Individuals are willing to use ICT technology for specific purposes, such as acquiring information, interacting with others, or utilizing it in their educational activities. A prior study^71^ supported our argument that higher TSE may lead to greater perceived trust in technology. Greater perceived trust in technology leads to individuals’ actual ICT use in their education. Building trust can involve ensuring the technology is secure, reliable, user-friendly, and capable of meeting the specific needs of the educational context^72^. The findings imply that trust promotes the actual use of ICT in Chinese education.

Secondly, perceived trust mediates the relationship between technological self-efficacy and the intention to use ICT tools for information. Individuals with higher TSE often have greater trust in their abilities to use technology effectively. This self-trust can extend to trust in the technology itself, as individuals believe they can overcome any potential challenges or issues arising during technology use^73^. TSE can increase the perceived usefulness of ICT tools for information about individuals' educational goals. Educational institutions in China should invest in training and support programs to enhance the technological self-efficacy of educators and students^74^. This will empower them to use ICT tools effectively. Individuals who trust the ICT technology are more likely to trust the information it delivers, leading to a higher intention to use these tools for acquiring information. Perceived trust is not limited to the functionality of ICT technology but also includes concerns about security and privacy^75^. Institutions and technology providers should build trust by ensuring ICT tools’ reliability, security, and effectiveness. Transparent communication about data handling and privacy policies can contribute to trust-building^76^.

Thirdly, perceived trust mediates the relationship between technological self-efficacy and the intention to use ICT tools for interaction. Educators and students with higher levels of TSE may be more willing to use ICT tools if they trust that they are reliable, secure, and credible. Trust in technology is critical because it reduces perceived risks and enhances the perceived benefits of technology adoption^77^. Educational institutions and technology providers should prioritize building trust in their ICT tools. This can be achieved through transparent communication about the security and privacy measures in place, ensuring consistent reliability, and providing user training and support^78^. Trust-building initiatives can contribute to a positive environment for the integration of ICT in education. It is essential to recognize that perceived trust is influenced not only by the inherent characteristics of ICT tools but also by external factors such as media reports, user experiences, and institutional policies^79^. Therefore, efforts to enhance perceived trust should encompass technological and environmental aspects. Recognizing the importance of perceived trust and improving it can foster a more conducive environment for successfully adopting and utilizing ICT tools in education, ultimately benefiting educators and students^80^.

Fourthly, the findings elaborate that perceived security significantly moderates the relationship between technological self-efficacy and perceived trust. Perceived security pertains to the measures and mechanisms in place to protect the privacy and security of data and information within the education sector, especially in the context of ICT technology use. When individuals trust the security of their data, they are more willing to use ICT^27^. A past study^81^ focused on individuals with higher TSE may better understand security features and practices, leading to a more accurate perception of security. When individuals perceive a technology as secure, it positively influences their trust. A sense of security is a fundamental component of trust. Trust is likely to be eroded if technology is seen as a potential source of risk or insecurity^82^. High TSE can enhance an individual's ability to navigate security features and practices effectively, reinforcing their perception of security and trust. Organizations that want to foster trust in their technology offerings should consider improving the security of their products and services and providing users with the knowledge and skills necessary to use technology effectively^83^. Empowering users with TSE can enhance their perception of security and, in turn, build trust in the technology.

Fifthly, the findings reveal that electronic word of mouth significantly moderates the relationship between technological self-efficacy and perceived trust. The eWOM usually involves individuals sharing opinions, reviews, or recommendations about products, services, or, in this case, educational experiences through electronic means, such as social media, forums, or online reviews^84^. The eWOM acts as a bridge between technology self-efficacy and perceived trust. When students and parents with high TSE share positive experiences and recommendations about digital learning tools or online educational platforms, it can enhance the perceived trust of others in these tools or platforms^85^. The eWOM also contributes to perceived trust by serving as a source of trustworthy information. In the Chinese education sector, eWOM can be a conduit between individuals’ TSE and their perceived trust in digital learning tools, platforms, and educational institutions^86^. Trustworthy and credible electronic word of mouth can help validate technology competence and encourage the adoption of digital learning solutions. However, ensuring the accuracy and reliability of eWOM is essential to maintaining trust in the education sector^87^.

### Study implications

This study has some important implications in the education sector of China. Firstly, this study's findings highlight the importance of promoting TSE among educators and stakeholders in China’s education sector. By providing training, support, and resources to boost individuals' confidence in using ICT tools, educational institutions can potentially increase the adoption and effective utilization of technology for information and interaction. Secondly, this study underscores the role of trust as a facilitator of technology adoption. Trust in technology systems and providers can positively influence educators’ willingness to embrace ICT for educational purposes. Educational institutions and policymakers should prioritize building trust in technology platforms through transparent and reliable data security measures, privacy protections, and clear communication strategies. Thirdly, the study’s findings emphasize the significance of security measures in fostering technology adoption. A secure digital environment ensures educators and students feel safe using ICT tools. Educational institutions should invest in robust cybersecurity infrastructure, policies, and practices to protect sensitive data and maintain the trust of students. Fourthly, eWOM influences individuals' perceptions and decisions regarding ICT adoption. Educational institutions can harness the power of eWOM by encouraging satisfied users to share their positive experiences and recommendations with colleagues, thereby creating a culture of peer support for technology integration. Lastly, this study suggests that by focusing on TSE, trust-building, security measures, and leveraging eWOM, China’s education sector can effectively promote the adoption and successful utilization of ICT for information and interaction, ultimately enhancing the quality of education in the digital age.

This study provides some contributions and potential guiding strategies for ICT users. First, this study helps understand educators' confidence in using technology, which is crucial for effectively incorporating ICT tools in education. This also offers training programs and workshops to improve students’ skills and confidence in using ICT tools. Second, exploring the role of perceived trust in technology adoption, this study highlights the importance of reliable ICT infrastructure and platforms in the education sector. Similarly, universities create a supportive environment where educators feel encouraged to experiment with new technologies without fear of failure. Third, this study emphasizes the role of security in influencing educators' willingness to adopt and use ICT tools, highlighting the need for robust cybersecurity measures in educational technology. Universities provide a secure and reliable ICT infrastructure to build trust among students. Fourth, this study examines the influence of eWOM on technology adoption, indicating the power of peer recommendations and reviews in shaping educators' perceptions and decisions. Foster a community of students who can share their positive experiences and recommendations about ICT tools. Finally, universities collaborate with ICT industry partners to co-develop and customize educational technologies that meet the specific needs of the Chinese education sector. This will ultimately contribute to improving the quality of education and preparing students for the digital age.

### Conclusion

The integration of ICT in the education sector has become a prominent trend, both globally and specifically in China. ICT tools, such as online learning platforms, virtual classrooms, and educational apps, have transformed how education is delivered and experienced. This transformation is particularly relevant in China, where the education sector has been rapidly evolving to keep pace with technological advancements and the demands of a digital age. The current research study has examined the relationship between TSE and intention to use ICT for information and interaction, as well as their actual use through perceived trust in the education sector of China. Furthermore, this study investigates the moderating role of perceived security and eWOM between TSE and perceived trust. This study has five different findings: Firstly, it established that the relationship between TSE and the actual utilization of ICT tools is substantially mediated by perceived trust. Second, the association between TSE and intention to use ICT tools for information is significantly mediated by perceived trust. Third, the association between TSE and intention to use ICT for interaction is significantly mediated by perceived trust. Fourth, the association between TSE and perceived trust is significantly moderated by perceived security. Fifth, the connection between TSE and perceived trust is strongly moderated by eWOM. By critically examining these factors, this study aims to provide valuable insights that can inform strategies for harnessing the full potential of ICT in Chinese education, ultimately contributing to the nation's broader goals of educational excellence and global competitiveness.

### Limitations and future directions

Despite making significant contributions, this study has some limitations that will allow other studies to explore the underlying subject. Firstly, our study may have limited generalizability due to its focus on the education sector in China. Future studies would consider that different countries and regions may have unique sociocultural, economic, and regulatory factors that influence the relationship. Secondly, our study, which was conducted at a specific time, relied on cross-sectional data. For future perspectives, researchers will use longitudinal research designs that could provide more robust evidence of causality and changes in the relationship over time. Thirdly, this study may rely on self-reported participant data, which can be subject to biases such as social desirability bias. Future research could incorporate objective measures of TSE and ICT use to reduce the potential for response bias. Fourthly, this study focused on factors like perceived trust and security that are important in ICT adoption. Future research could benefit from more objective measures, such as cybersecurity assessments and audits, to accurately evaluate trust and safety in ICT systems. Lastly, this study’s moderate variable, eWOM, may not fully capture the nuances of online discussions and recommendations. Future research could explore more comprehensive methods for assessing eWOM, such as natural language processing techniques to analyze online content. Additionally, future researchers should adapt to the evolving landscape of technology to determine the relevance and applicability of self-efficacy, trust, security, and eWOM in emerging educational technologies.

### Ethics statements

We confirm that all methods were carried out in accordance with relevant guidelines and regulations. Humans who participated in this study are aware of the purpose of the study, and their confidential information is not to be shared with anyone. All study participants provided their written informed consent. Study data is used after the consent of participants. The questionnaire used in this study started with the declaration and purpose of the study. Experimental protocol was approved by the ethical review board of the Beijing University of Technology.

## Data Availability

The raw data supporting the conclusions of this article will be made available by the authors, without undue reservation. Data can be obtained upon reasonable request by Co-author, Muhammad Farrukh Shahzad (farrukhshahzad207@gmail.com).
